# Supplementary material for: Economic impact of the first wave of the COVID-19 pandemic on acute care hospitals in Japan
Source: PLoS One. 2020 Dec 31;15(12):e0244852. doi: 10.1371/journal.pone.0244852 (PMC7775082; doi:10.1371/journal.pone.0244852)
Supplement: S8 Fig — (DOCX) [file pone.0244852.s008.docx]

Figure S8. Year-over-year comparisons of the number of cases and hospital charges according to the patients’ ages.
