# Supplementary material for: Economic impact of the first wave of the COVID-19 pandemic on acute care hospitals in Japan
Source: PLoS One. 2020 Dec 31;15(12):e0244852. doi: 10.1371/journal.pone.0244852 (PMC7775082; doi:10.1371/journal.pone.0244852)
Supplement: S9 Fig — (DOCX) [file pone.0244852.s009.docx]

Figure S9. Year-over-year comparisons of the number of cases and hospital charges for inpatients in hospitals with and without COVID-19 patients.
