# Supplementary material for: Economic impact of the first wave of the COVID-19 pandemic on acute care hospitals in Japan
Source: PLoS One. 2020 Dec 31;15(12):e0244852. doi: 10.1371/journal.pone.0244852 (PMC7775082; doi:10.1371/journal.pone.0244852)
Supplement: S1 Table — (DOCX) [file pone.0244852.s013.docx]

Table S1. Characteristics of hospitals for the study (comparisons of before and after the state of emergency)

| Variable | Before | | After | |
| --- | --- | --- | --- | --- |
|  | (July 2018 to March 2020) | | (April to May 2020) | |
| Number of monthly cases per hospital | | | | |
| Inpatients |  |  |  |  |
| Mean ± SD | 598.9 | ± 442.7 | 480.0 | ± 357.5 |
| Median (1Q, 3Q) | 468 | (261, 827) | 377 | (211, 656) |
| Urgent admission |  |  |  |  |
| Mean ± SD | 303.3 | ± 207.9 | 250.6 | ± 174.5 |
| Median (1Q, 3Q) | 244 | (140, 421) | 199 | (113, 342) |
| Admission with surgery |  |  |  |  |
| Mean ± SD | 263.6 | ± 223.2 | 215.4 | ± 185.5 |
| Median (1Q, 3Q) | 189 | (104, 359) | 159 | (83, 291) |
| Outpatients |  |  |  |  |
| Mean ± SD | 11685.5 | ± 8431.1 | 9156.2 | ± 6603.6 |
| Median (1Q, 3Q) | 9470 | (6294, 14971) | 7322 | (4730, 11886) |
| Monthly hospital charges per hospital (million Japanese Yen) | | | | |
| Sum of inpatients and outpatients | | |  |  |
| Mean ± SD | 746.9 | ± 650.1 | 666.4 | ± 585.3 |
| Median (1Q, 3Q) | 516 | (320, 949) | 466 | (288, 849) |
| Inpatients |  |  |  |  |
| Mean ± SD | 520.4 | ± 434.9 | 467.3 | ± 388.3 |
| Median (1Q, 3Q) | 375 | (224, 671) | 337 | (199, 593) |
| Outpatients |  |  |  |  |
| Mean ± SD | 226.5 | ± 233.0 | 199.1 | ± 214.8 |
| Median (1Q, 3Q) | 151 | (87, 282) | 132 | (71, 242) |

SD, standard deviation; 1Q, 1st quartile; 3Q, 3rd quartile.
