# Supplementary material for: Economic impact of the first wave of the COVID-19 pandemic on acute care hospitals in Japan
Source: PLoS One. 2020 Dec 31;15(12):e0244852. doi: 10.1371/journal.pone.0244852 (PMC7775082; doi:10.1371/journal.pone.0244852)
Supplement: S2 Table — Characteristics of the study population by inpatients and outpatients a. Inpatients. (DOCX) [file pone.0244852.s014.docx]

Table S2. Characteristics of the study population by inpatients and outpatients

a. Inpatients

| Variable | Mean ± SD, median (1Q, 3Q), or n (%) | |
| --- | --- | --- |
| Number of cases | 2,739,878 |  |
| Sex |  |  |
| Male | 1,444,673 | (52.7%) |
| Age |  |  |
| Mean ± SD | 63.5 | ± 24.0 |
| Median (1Q, 3Q) | 71 | (53, 80) |
| Category |  |  |
| –17 | 215,882 | (7.9%) |
| 18–64 | 793,509 | (29.0%) |
| 65– | 1,730,487 | (63.2%) |
| Urgent admission | 1,391,213 | (50.8%) |
| Admission with surgery | 1,209,073 | (44.1%) |
| Hospital charges per case (million Japanese Yen) | |  |
| Mean ± SD | 878,352 | ± 1,182,174 |
| Median (1Q, 3Q) | 527,604 | (245142, 1055375) |
| Length of hospital stay (day) |  |  |
| Mean ± SD | 15 | ± 25.8 |
| Median (1Q, 3Q) | 8 | (4, 16) |

b. Outpatients

| Variable | Mean ± SD, median (1Q, 3Q), or n (%) | |
| --- | --- | --- |
| Number of cases | 53,479,658 |  |
| Sex |  |  |
| Male | 26,488,867 | (49.5%) |
| Age |  |  |
| Mean ± SD | 60.9 | ± 22.0 |
| Median (1Q, 3Q) | 68 | (50, 77) |
| Category |  |  |
| –17 | 3,857,731 | (7.2%) |
| 18–64 | 19,539,647 | (36.5%) |
| 65– | 30,082,280 | (56.2%) |
| Hospital charges per case (million Japanese Yen) | |  |
| Mean ± SD | 19,606 | ± 63,260 |
| Median (1Q, 3Q) | 8,060 | (3020, 20980) |

SD, standard deviation; 1Q, 1st quartile; 3Q, 3rd quartile.
