# Supplementary material for: Economic impact of the first wave of the COVID-19 pandemic on acute care hospitals in Japan
Source: PLoS One. 2020 Dec 31;15(12):e0244852. doi: 10.1371/journal.pone.0244852 (PMC7775082; doi:10.1371/journal.pone.0244852)
Supplement: S3 Table — (DOCX) [file pone.0244852.s015.docx]

Table S3. Characteristics of the study population by inpatients and outpatients (comparisons of before and after the state of emergency)

a. Inpatients

| Variable | Before | | After | |
| --- | --- | --- | --- | --- |
|  | (July 2018 to March 2020) | | (April to May 2020) | |
| N | 2,452,529 |  | 187,190 |  |
| Sex |  |  |  |  |
| Male | 1,292,266 | (52.7%) | 98,972 | (52.9%) |
| Age |  |  |  |  |
| Mean ± SD | 63.3 | ± 24.2 | 65.3 | ± 22.4 |
| Median (1Q, 3Q) | 70 | (53, 80) | 71 | (56, 81) |
| Category |  |  |  |  |
| –17 | 199,806 | (8.1%) | 10,457 | (5.6%) |
| 18–64 | 709,623 | (28.9%) | 54,781 | (29.3%) |
| 65– | 1,543,100 | (62.9%) | 121,952 | (65.1%) |
| Urgent admission | 1,241,848 | (50.6%) | 97,744 | (52.2%) |
| Admission with surgery | 1,079,599 | (44.0%) | 84,003 | (44.9%) |
| Hospital charges per case (million Japanese Yen) | | |  |  |
| Mean ± SD | 868,960 | ± 1,167,506 | 973,584 | ± 1,282,098 |
| Median (1Q, 3Q) | 520,827 | (241560, 1042586) | 597,804 | (285803, 1187393) |
| Length of hospital stay (day) | | |  |  |
| Mean ± SD | 15 | ± 25.6 | 16 | ± 28.4 |
| Median (1Q, 3Q) | 8 | (4, 16) | 9 | (4, 18) |

*(continued)*

Table S3. *(continued)*

b. Outpatients

| Variable | Before | | After | |
| --- | --- | --- | --- | --- |
|  | (July 2018 to March 2020) | | (April to May 2020) | |
| N | 47,851,971 |  | 3,570,926 |  |
| Sex |  |  |  |  |
| Male | 23,647,550 | (49.4%) | 1,819,307 | (50.9%) |
| Age |  |  |  |  |
| Mean ± SD | 60.9 | ± 0.0 | 60.9 | ± 0.0 |
| Median (1Q, 3Q) | 68 | (0, 0) | 68 | (0, 0) |
| Category |  |  |  |  |
| –17 | 3,565,043 | (7.5%) | 179,368 | (5.0%) |
| 18–64 | 17,490,040 | (36.6%) | 1,303,849 | (36.5%) |
| 65– | 26,796,888 | (56.0%) | 2,087,709 | (58.5%) |
| Hospital charges per case (million Japanese Yen) | | | |  |
| Mean ± SD | 19,384 | ± 61,777 | 21,747 | ± 75,932 |
| Median (1Q, 3Q) | 8,020 | (3010, 20800) | 8,450 | (2980, 23220) |

SD, standard deviation; 1Q, 1st quartile; 3Q, 3rd quartile.
