# Supplementary material for: Economic impact of the first wave of the COVID-19 pandemic on acute care hospitals in Japan
Source: PLoS One. 2020 Dec 31;15(12):e0244852. doi: 10.1371/journal.pone.0244852 (PMC7775082; doi:10.1371/journal.pone.0244852)
Supplement: S4 Table — (DOCX) [file pone.0244852.s016.docx]

Table S4. The Number and cumulative LOS of COVID-19 cases

|  | All | | | Confirmed COVID-19 cases | | | Suspected COVID-19 cases | | |
| --- | --- | --- | --- | --- | --- | --- | --- | --- | --- |
| Month | Number of hospitals | Number of cases | Sum of LOS (day) | Number of hospitals | Number of cases | Sum of LOS (day) | Number of hospitals | Number of cases | Sum of LOS (day) |
| Jan 2020 | 3 | 3 | 9 | 0 | 0 | 0 | 3 | 3 | 9 |
| Feb 2020 | 25 | 57 | 434 | 7 | 16 | 200 | 23 | 42 | 242 |
| Mar 2020 | 70 | 338 | 3,831 | 33 | 115 | 1,746 | 62 | 225 | 2,119 |
| Apr 2020 | 147 | 1,420 | 16,282 | 93 | 513 | 6,068 | 134 | 923 | 10,343 |
| May 2020 | 160 | 2,652 | 38,912 | 87 | 451 | 9,080 | 153 | 2,209 | 29,990 |
| Jun 2020 | 150 | 2,980 | 47,438 | 49 | 150 | 3,675 | 149 | 2,834 | 43,897 |

LOS, length of hospital stay.
